# From Kidneys to Nerves: A Rare Case of a Urinary Tract Infection Triggering Guillain-Barré Syndrome

**DOI:** 10.7759/cureus.113521

**Published:** 2026-07-28

**Authors:** Molly VanLuling, Nicole Buleza

**Affiliations:** 1 Family Medicine, University of Massachusetts Chan Medical School, Worcester, USA; 2 Internal Medicine, Inspira Health Network - Mullica Hill, Mullica Hill, USA

**Keywords:** ascending flaccid paralysis, e. coli, e. coli bacteremia, guillain-barré syndrome variants, urinary tract infection

## Abstract

Guillain-Barré syndrome (GBS) is an autoimmune disease typically caused by an acute viral illness that can lead to ascending paralysis. Rarely, GBS has been reported following an *Escherichia coli* urinary tract infection (UTI). A 68-year-old female with multiple comorbidities presented to the hospital for acute bilateral lower extremity weakness. She had been treated for a UTI one week prior. Clinical and radiologic evaluation were highly suggestive of GBS, although the patient declined confirmatory lumbar puncture. The patient recovered lower extremity function following a five-day course of intravenous immunoglobulin. Most interestingly, the patient returned to the emergency department on the day of discharge for chills, dizziness, and nausea and was found to have recurrent versus inadequately treated *E. coli* UTI. This case illustrates the importance of diagnostic consideration for infections less commonly associated with GBS. Additionally, the case highlights the need for concurrent treatment of ongoing infectious processes in addition to recommended immunotherapy.

## Introduction

Guillain-Barré syndrome (GBS) is a rare autoimmune disease characterized by the abrupt onset of lower extremity weakness leading to ascending paralysis, often following acute respiratory or gastrointestinal illness. Common causes of GBS include *Campylobacter jejuni* infection, cytomegalovirus, Epstein-Barr virus, and *Mycoplasma pneumoniae* [1]. The immunologic process for paralysis as a result of GBS is not well understood, though *C. jejuni* is known to imitate peripheral nerve gangliosides, a mechanism that is thought to be similar in other pathogens [2]. Due to this imitation, the body attacks the gangliosides, leading to peripheral paralysis. Much less commonly, GBS is triggered by a urinary tract infection (UTI) [1,3]. The following case presents a 68-year-old female with sudden lower extremity weakness following a recent *Escherichia coli* UTI and presumed GBS. As UTIs are an uncommon cause of GBS, this case highlights the importance of screening for and treating UTIs when evaluating patients with GBS, particularly in the setting of other potential infectious triggers.

## Case presentation

A 68-year-old female with a history of type 2 diabetes, treated hepatitis C, heart failure with reduced ejection fraction, and a recent UTI one week prior presented with generalized weakness and inability to walk that started suddenly while grocery shopping. The patient described herself as usually very active, including even dancing at a wedding two weeks prior. She reported significant nausea and vomiting one week prior. Although gastroenteritis was an initial outpatient consideration, at that time she was found to have a UTI and was treated empirically with a one-time dose of fosfomycin. While her nausea and vomiting had improved, generalized weakness persisted. Urine culture later resulted in >100,000 colony-forming units (CFU)/mL *E. coli*. On admission, the patient’s vital signs were within normal limits. Neurological examination showed 2/4 patellar reflexes bilaterally and decreased 1/5 muscle strength in the lower extremities bilaterally with intact sensation. The patient had no upper extremity or other neurologic deficits. She had no shortness of breath or extremity edema. Erythrocyte sedimentation rate, C-reactive protein, creatine kinase, and thyroid-stimulating hormone were all within normal limits (Table 1). B-type natriuretic peptide was 6 pg/mL. Urine drug screen, COVID-19, influenza, and respiratory syncytial virus testing were all negative (Table 2).

**Table 1:** Completed blood work during hospitalization. WBC: white blood cell; ESR: erythrocyte sedimentation rate; CRP: C-reactive protein; PT: prothrombin time; INR: international normalized ratio; TSH: thyroid-stimulating hormone; CK: creatine kinase; UDS: urine drug screen

	Day 1	Day 2	Day 3	Day 4	Day 6	Day 8	Reference
WBC	6.4	5.7	5.8	4.0	3.9	5.8	4.0–11.0 × 10³/µL
Hemoglobin	16.6	15.5	14.6	14.3	14.3	13.7	11.0–15.2 g/dL
Platelet	261	228	216	195	193	158	140–380 × 10³/µL
ESR	17	-	-	-	-	-	0–30 mm
CRP	<0.5	-	-	-	-	-	0.1–0.9 mg/dL
PT	-	12.0	-	-	-	-	9.7–13.3 seconds
INR	-	1.0	-	-	-	-	0.8–1.2
Creatinine	1.61	1.5	1.14	1.12	1.17	1.23	0.7–1.3 mg/dL
TSH	1.99	-	-	-	-	-	0.550–4.78 U/L
CK	96	-	-	-	-	-	46–171 U/L
UDS	Negative	-	-	-	-	-	Negative

**Table 2:** Urinalysis and viral testing results. UA: urinalysis; RSV: respiratory syncytial virus

Lab test	Day 1	Day 8	Reference
UA	4+ bacteria, positive nitrites, large leukoesterase	4+ bacteria, positive nitrites, large leukoesterase	Negative bacteria, negative nitrites, negative leukoesterase
COVID-19	Negative	-	Negative
RSV	Negative	-	Negative
Influenza	Negative	-	Negative

**Figure 1:**
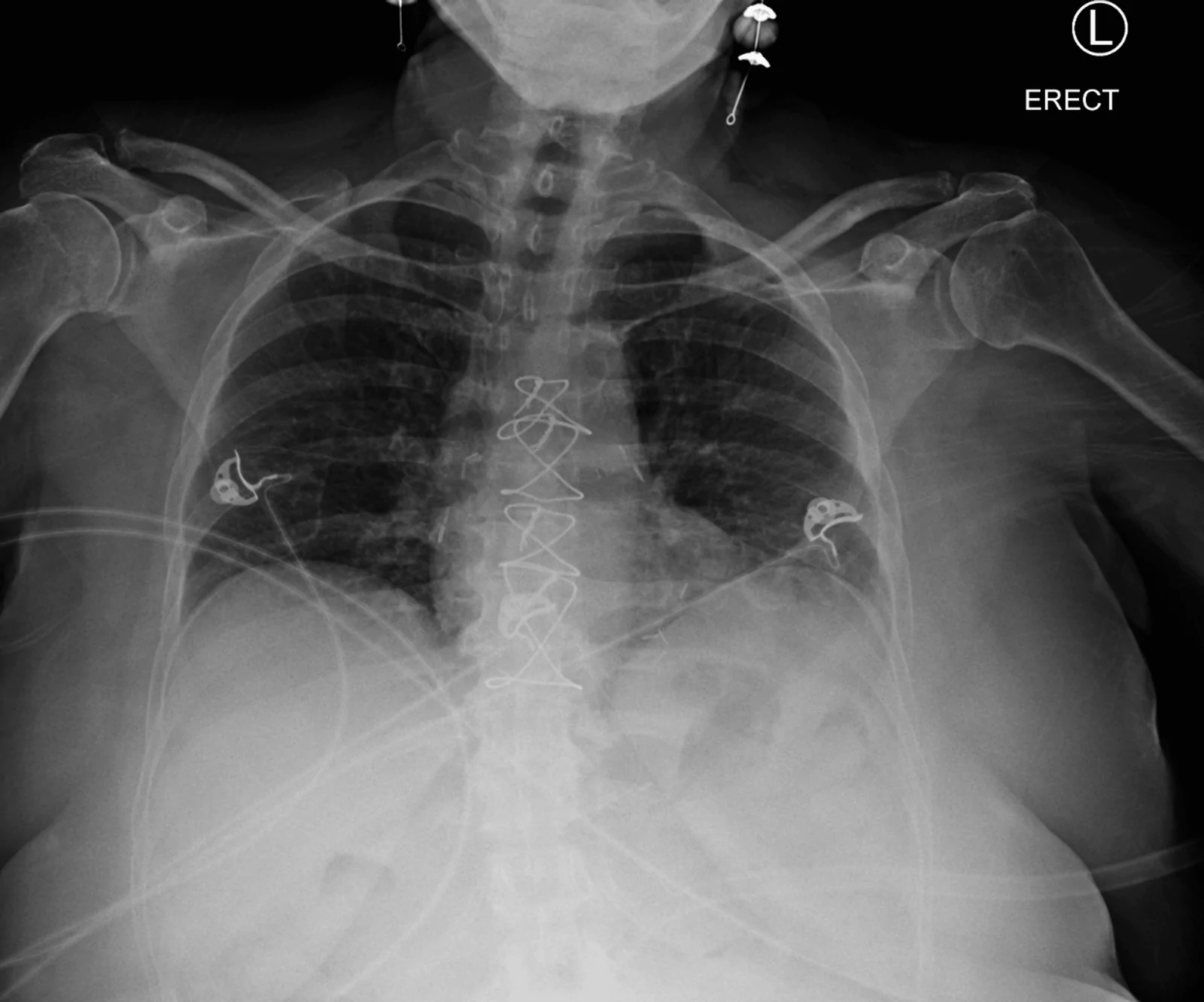
Chest X-ray. Negative chest X-ray showing clear lungs.

**Figure 2:**
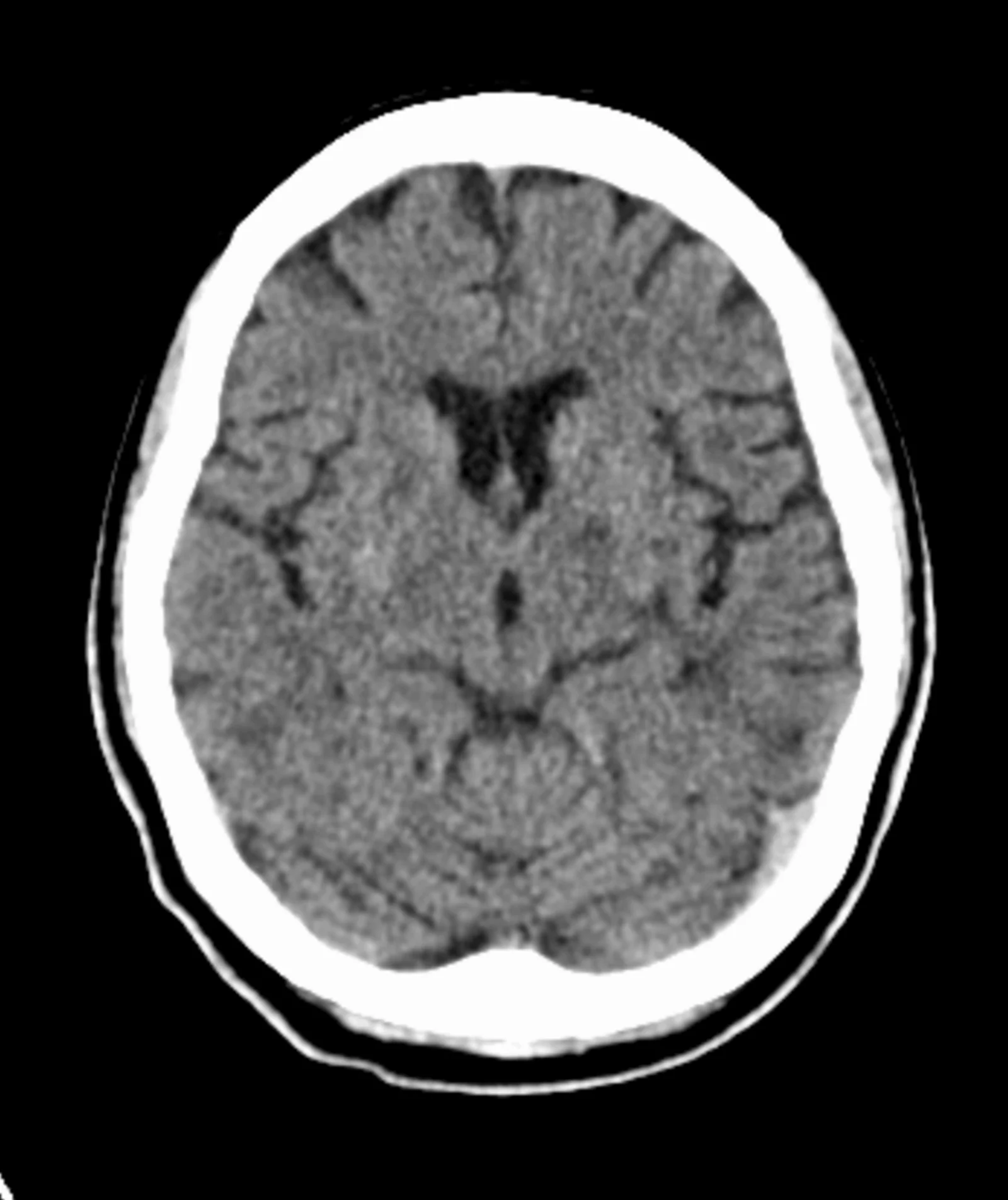
Head CT. Head CT scan with no acute intracranial abnormality. Mild cerebral atrophy can be noted.

**Figure 3:**
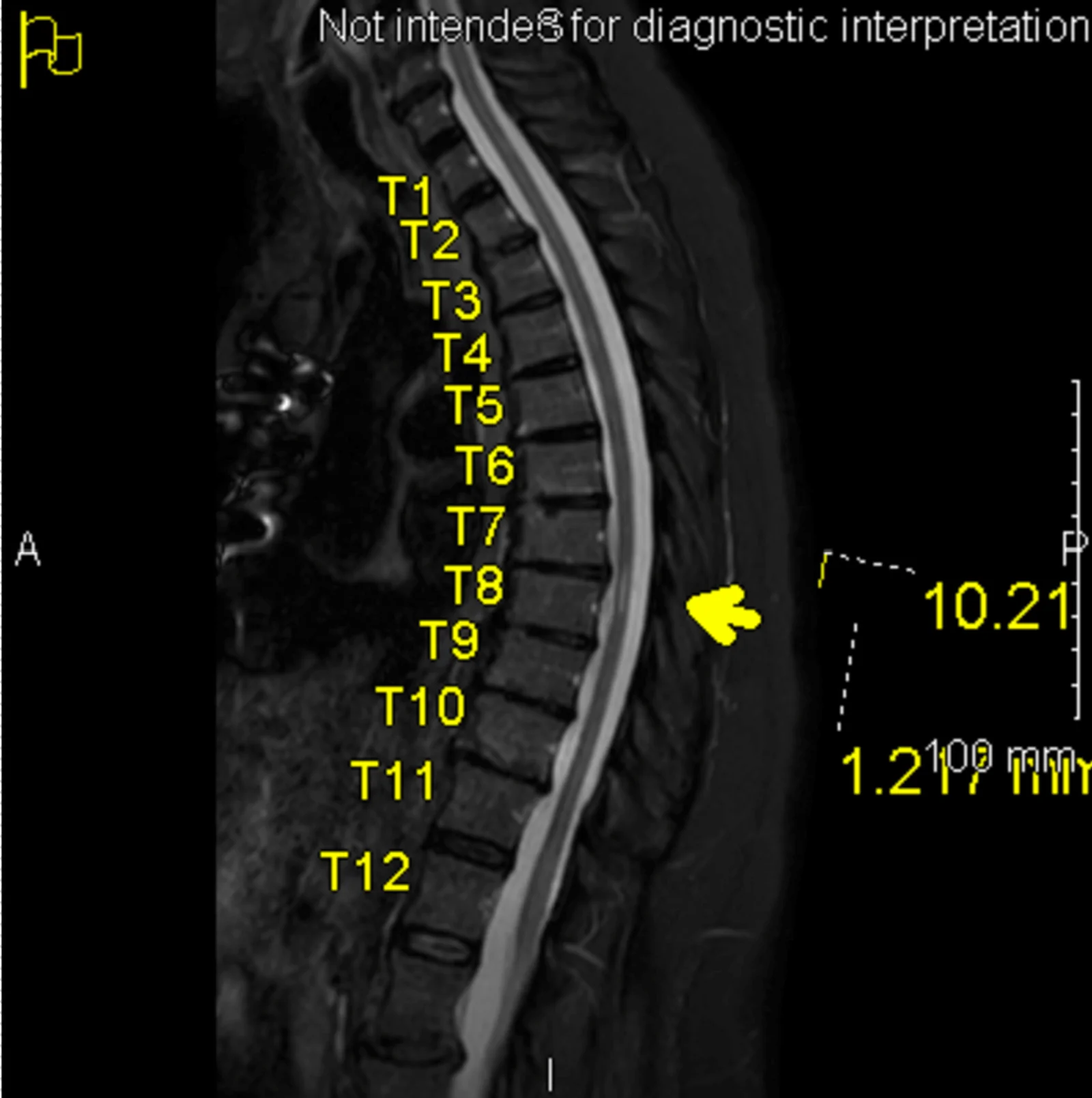
MRI of the thoracic spine (sagittal view). Sagittal view of MRI of the thoracic spine with and without contrast showing linear area of T2 hyperintensity in the thoracic spinal cord at T7-8 through mid T8 level measures up to 10 mm craniocaudal length × 1.2 mm. Slight cord thinning can be noted in this region.

**Figure 4:**
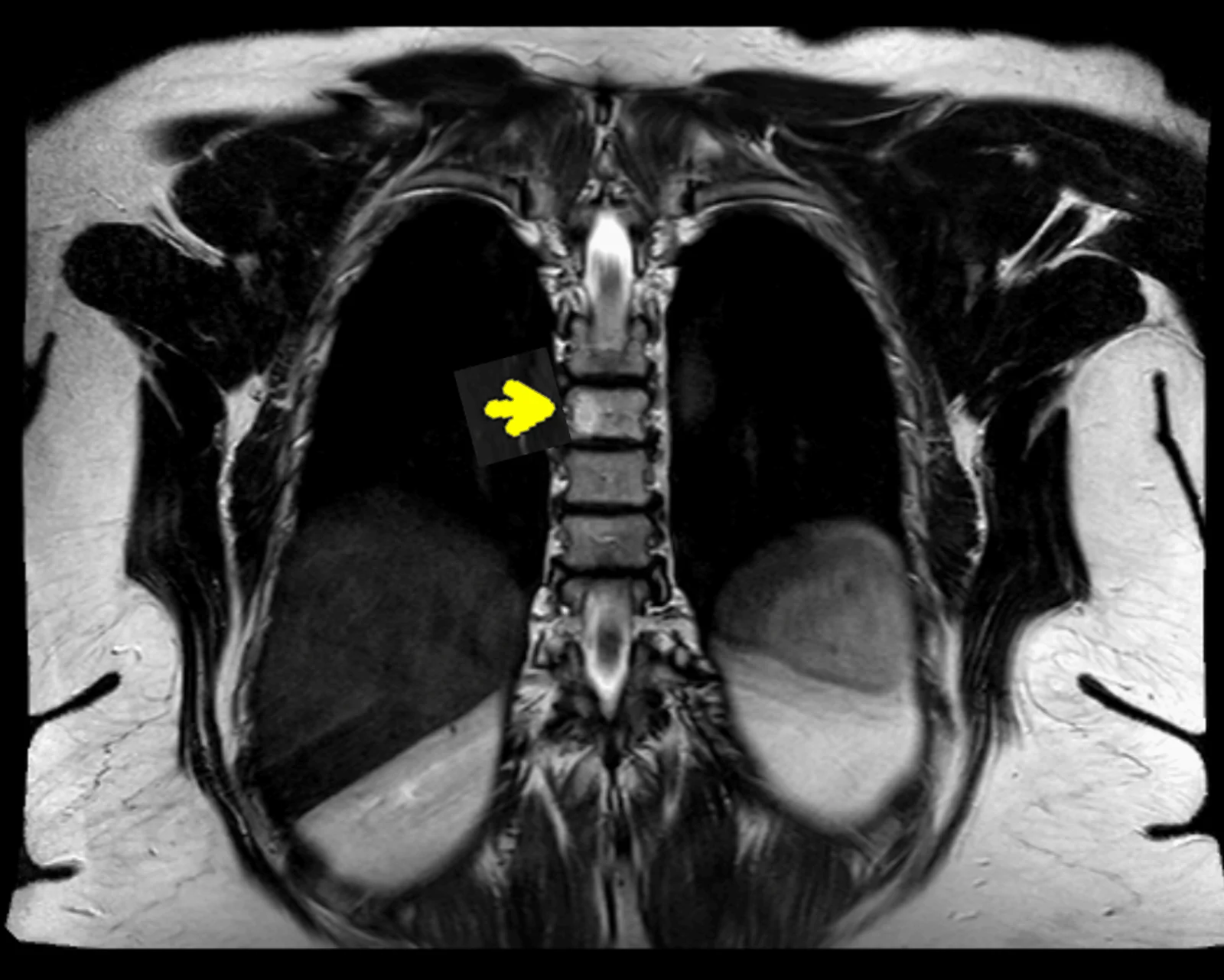
MRI of the thoracic spine (coronal view). Coronal view of MRI of the thoracic spine with and without contrast showing linear T2 hyperintensity in the thoracic spinal cord at T7-8. The signal abnormality is slightly more eccentric to the left. Demyelinating disease would be in the differential.

Neurology was consulted due to concerns for possible GBS. Chest X-ray showed clear lungs (Figure 1). Head CT showed mild cerebral atrophy without acute intracranial abnormalities (Figure 2). Cervical, thoracic, and lumbar MRIs were ordered and showed no acute pathology, although hyperintensities with slight cord thinning were visualized on thoracic MRI potentially suggestive of a demyelinating process (Figures 3, 4).

Unfortunately, the patient declined lumbar puncture. Electroneuromyography was not performed during her stay as the hospital was limited in its ability to provide this inpatient. She was started on empiric treatment for GBS with a five-day course of intravenous immunoglobulin at a total dose of 2 g/kg divided over the five days. She showed improvement in muscle strength within two days of treatment to 4/5 in the lower extremities bilaterally. She continued to have no strength deficits in the upper extremities bilaterally. She was hospitalized for a total of eight days, complicated by continued nausea treated with intravenous ondansetron and increasing back spasms treated with cyclobenzaprine, before she was discharged to subacute rehabilitation. The same day of discharge, she returned to the emergency department for chills, dizziness, and nausea. Her urinalysis was again consistent with a UTI, showing 4+ bacteria, positive nitrites, and large leukoesterases. Ceftriaxone 1 g intravenous was administered, and the patient was discharged to subacute rehabilitation with a prescription for cefdinir 300 mg q12 hours for five days. Urine culture again resulted in >100,000 CFU/mL *E. coli*.

## Discussion

Pyelonephritis in the setting of *E. coli* UTI represents an exceedingly rare trigger for GBS. Though overt kidney infection was not diagnosed in this case, persistent UTI due to *E. coli* was definitively present. While molecular mimicry between bacterial surface antigens and peripheral nerve gangliosides is well-established as a mechanism for *C. jejuni*, the mechanism by which *E. coli* might trigger GBS remains poorly understood [1,3]. The timing of this patient’s UTI one week before symptom onset falls within the typical one- to four-week window for infection-triggered GBS [3,4]. Jo et al. [1] reported a similar case of recurrent GBS following *E. coli* UTI, suggesting that *E. coli*-induced UTI may be an underrecognized trigger involved in GBS. Furthermore, previous research has shown that fosfomycin may be an inadequate treatment for UTI caused by Gram-negative organisms due to its low serum concentration and therefore lower efficacy in patients with bacteremia or pyelonephritis [5]. *E. coli* is implicated as the pathogenic cause of UTI in 80-85% of cases, increasing its significance for consideration in GBS workup due to its frequency of occurrence [6].

Recognition of these atypical presentations is essential, as they may constitute nearly half of GBS cases in some populations [7]. However, weighted consideration needs to be given to other potential diagnoses that could lead to lower extremity weakness and paralysis. Confirmatory diagnosis with lumbar puncture or testing by electroneuromyography could have pointed to a more certain GBS diagnosis. Unfortunately, these tests were not performed. The patient’s paralysis also never progressed to the upper extremities or inhibited her respiratory ability. Though the thoracic MRI findings of hyperintensities with cord thinning excluded diagnoses such as myelopathy or cauda equina syndrome, the MRI was non-diagnostic and did not rule out demyelinating pathology such as multiple sclerosis [3].

With these considerations taken as a whole, the patient’s clinical presentation was initially puzzling. Her symmetric lower extremity paralysis was not characteristic of multiple sclerosis. Despite a lack of upper extremity symptoms, she had improvement in both strength and movement within two days of intravenous immunoglobulin initiation. It was debated whether conversion syndrome should be considered as a diagnosis given the patient’s extremely rapid improvement (particularly because this was before receiving a full antibiotic course for the apparently causative UTI), but no recent stressful event could be elucidated from the patient or her spouse. Although rapid, her symptom improvement did align with the anticipated therapeutic outcome of intravenous immunoglobulin use in GBS, and her *E. coli* UTI was likely the initiating factor due to being inadequately treated with fosfomycin [5,8]. This case underscores that GBS remains a clinical diagnosis requiring pattern recognition rather than strict adherence to diagnostic criteria.

## Conclusions

This case presents a rare manifestation of suspected GBS triggered by *E. coli*-induced UTI in a 68-year-old female with multiple comorbidities. The patient’s hospital course further highlights the importance of a complete workup in the care of a patient with GBS. Our patient had initial symptoms of nausea and vomiting and was presumed to have GBS as a result of gastroenteritis. Empiric immunotherapy with intravenous immunoglobulin is appropriate when clinical presentation is compelling, even when diagnostic confirmation (such as a causative bacterial or viral organism) is limited. The favorable response to treatment supports early intervention in suspected GBS. However, while her symptoms were treated with intravenous immunoglobulin, her incompletely treated UTI was overlooked, triggering what was thought to be a continued autoimmune response. Due to the high prevalence of *E. coli* as a source of UTIs and its relevance as a source for GBS, we encourage providers to add urinalysis testing to their workup for GBS to provide a comprehensive picture of possible pathogenic causes beyond respiratory or gastrointestinal sources. In considering UTIs as a potential trigger for GBS, recurrence of symptoms may be prevented. Further investigation into the mechanisms by which urinary pathogens, such as *E. coli*, trigger autoimmune polyradiculoneuropathies may improve risk stratification and prevention strategies.
